# A hexapod walker using a heterarchical architecture for action selection

**DOI:** 10.3389/fncom.2013.00126

**Published:** 2013-09-17

**Authors:** Malte Schilling, Jan Paskarbeit, Thierry Hoinville, Arne Hüffmeier, Axel Schneider, Josef Schmitz, Holk Cruse

**Affiliations:** ^1^Center of Excellence ‘Cognitive Interaction Technology,’ Bielefeld UniversityGermany; ^2^Biological Cybernetics, Bielefeld UniversityGermany

**Keywords:** insect locomotion, motor control, decentral architecture, action selection

## Abstract

Moving in a cluttered environment with a six-legged walking machine that has additional body actuators, therefore controlling 22 DoFs, is not a trivial task. Already simple forward walking on a flat plane requires the system to select between different internal states. The orchestration of these states depends on walking velocity and on external disturbances. Such disturbances occur continuously, for example due to irregular up-and-down movements of the body or slipping of the legs, even on flat surfaces, in particular when negotiating tight curves. The number of possible states is further increased when the system is allowed to walk backward or when front legs are used as grippers and cannot contribute to walking. Further states are necessary for expansion that allow for navigation. Here we demonstrate a solution for the selection and sequencing of different (attractor) states required to control different behaviors as are forward walking at different speeds, backward walking, as well as negotiation of tight curves. This selection is made by a recurrent neural network (RNN) of motivation units, controlling a bank of decentralized memory elements in combination with the feedback through the environment. The underlying heterarchical architecture of the network allows to select various combinations of these elements. This modular approach representing an example of neural reuse of a limited number of procedures allows for adaptation to different internal and external conditions. A way is sketched as to how this approach may be expanded to form a cognitive system being able to plan ahead. This architecture is characterized by different types of modules being arranged in layers and columns, but the complete network can also be considered as a holistic system showing emergent properties which cannot be attributed to a specific module.

## Introduction

In this article, we propose a simple neural architecture that consists of basically independent, parallel sensori-motor procedures—or modules—that allows to orchestrate these modules. In addition this architecture shows the property for easy expansions of the system. This architecture is not based on a specific biological brain structure, but is inspired by behavioral experiments on insects (Cruse et al., 2009a) as are walking on unpredictable environment, performed with stick insects, and navigation, performed with desert ants and honey bees. Nonetheless, such a modular architecture may be of broader interest because many authors assume that a modular structure is a basic property of brains in general.

For example, Anderson (2010) has argued that evolution had to find specific solutions for quite different requirements posed by specific environmental conditions as are locomotion, mating, navigation or feeding, and problems occurring later during evolutionary development may be solved by combining existing (functional) modules in different ways, following the principle of “neural reuse” (Anderson, 2010). In this way, different procedures might be developed that finally serve the same or very much related purposes, thus leading to redundant structures. In insect navigation, for example, path integration and landmark navigation are used in parallel. In this case, different input modules are used to drive the same output elements. In turn, the same motor structure may be used for different purposes. This is obvious in the case of the Praying Mantis, where front legs can be used for walking, but can also be used for a completely different purpose, namely for catching prey, which requires specific neuronal structures for these different functions. Furthermore, Flash and Hochner (2005) have reviewed results on both vertebrates and invertebrates that lead these authors to the interpretation that “many different movements can be derived from a limited number of stored primitives.” These movements can further be “combined through a well defined syntax of action to form more complex action.”

Therefore, many results suggest the existence of such discrete primitives. The question as to how behavioral choice is performed, i.e., how the lower level elements are selected and orchestrated to control a specific behavior is still open (Briggman and Kristan, 2008). Activating such a subfset means to select a specific internal state that sets priorities. At a higher level, this corresponds to the faculty of selective attention, i.e., the enactment of internal states that control which sensory input may be exploited. This top–down influence may be complemented by bottom–up attention: sufficiently strong, specific sensory inputs could influence and change the internal state of the system.

In this article, we propose a way how this problem could be addressed by exploiting an artificial neural network, Walknet, that has been developed to describe a huge amount of biological data concerning insect, i.e., hexapod (forward) walking (Dürr et al., 2004; Schilling et al., 2013). This network consists of a number of parallel modular elements able to control “microbehaviors” as for example swing movement of the left front leg or stance movement of the right middle leg. This decentralized architecture is able to control forward walking within a continuous range of velocities as observed in stick insects (Graham, 1972) or Drosophila (Wosnitza et al., 2013). Walknet can further describe a large number of behavioral experiments performed with stick insects, including difficult cases like climbing over a gap of about body size. In doing so, the controller shows its robustness with respect to disturbances resulting from unpredictable environment but also from the complex dynamics of the own body.

So, Walknet is able to deal with complex behavior, but still controls only one overarching specific context, namely forward walking as will be briefly reviewed in Section The Basic Version (for a recent review see Schilling et al., 2013). According to this structure, only local decisions are required, which concern the decision between swing and stance for a given leg. Walknet has been expanded by a body model, briefly reviewed in Section Body Model (for details see Schilling, 2011; Schilling et al., 2012), that represents the kinematics of the body, i.e., all 18 joints of the six legs plus two body joints, each with 2° of freedom, as can be found in stick insects and as they are used by the physical hexapod robot Hector (Schneider et al., 2011). However, to allow for controlling different behaviors, further decision structures are required. A simple case concerns the decision between forward and backward walking. In addition, the system may decide between 6-legged walking and 4-legged walking, where the front legs may be used for other purposes, for example as grippers, as is the case in the above mentioned Mantis. A more general problem concerns the ability to exhibit trial-and-error behavior, i.e., select a behavior that is not selectable in the current context. This faculty is a prerequisite for a further capability, namely being able to plan ahead (i.e., internal simulation of a selected behavior), a property which may not be found on the insect level. Another problem concerns the question how biological systems may be able to invent and deal with symbols, or new concepts. Hints exist that this property can already be found in insects (Giurfa et al., 2001), at least on a simple level.

Based on the already given Walknet, in this article we will introduce a neural architecture that provides a precondition for dealing with those problems. To this end, we will adopt a structure already successfully applied within a network, Navinet, that is able to control insect-like navigation (Cruse and Wehner, 2011; Hoinville et al., 2012). Navinet can then be used as an expansion of Walknet to allow for decisions at higher levels of integration. For example, a foraging ant may decide between selecting one of different food sources stored in memory, and, at a lower level, to select between inbound or outbound travels. After having selected the food source, the ant may decide to attend a visual landmark seen or not. In Navinet, this ability is given by a so-called motivation unit network, a structure that will now be applied to Walknet, too (Section Motivation Unit Network). This expansion allows the complete system to decide between different behaviors at different levels of integration. In the final version this will lead us to an architecture that will consist of many parallel “columns” which are organized in four layers. As the motivation unit network can be considered the “backbone” of the architecture proposed here, we call the latter Motivation Unit Based Columnar Architecture (MUBCA).

In this article we will show how Walknet can be expanded by motivation units and still does inherit its earlier properties. To this end the behavior of the expanded Walknet will be tested in different situations of forward walking including starting from uncomfortable leg configurations and the negotiation of very tight curves. These tests are critical for studying the stability of the system because due to the irregular stepping patterns, resulting from the dynamical properties of the body including possible leg slipping, the complete system is subject to continuous disturbances. We further will show that the network can select between states of forward walking and backward walking. To illustrate how the motivation unit network could be expanded for further procedures, as an example we will discuss the case of switching between 6-legged walking and 4-legged walking (Section Discussion) and will note how to connect Walknet with Navinet to equip the complete system with insect-like navigation procedures as are vector navigation and landmark navigation. In addition, to illustrate the capacity of this architecture we will briefly sketch how this structure could be exploited to equip the complete system with cognitive abilities in the sense to be able to plan ahead and how the use of symbols may be possible.

## Walknet

### The basic version

Tightly based on the morphology of a stick insect (*Carausius morosus*), the walker has six legs, each of which is equipped with three joints. The abdomen and the head of the insect are not functionally relevant for walking. Therefore, the body of the walker consists of only three functional relevant body segments. The leg pairs are connected to those three body segments. The body segments are connected by joints, each allowing for 2° of freedom (up and down as well as side movements of the body segments). Therefore, the controller has to deal with 22° of freedom (DoF). As the position of one body segment in space is defined by only six DoFs (three for position in space, three for orientation) there are 16 DoFs free to be decided upon by the controller. However, “free” does not mean that the controller may leave the decision open, rather it has to make these 22 decisions in a sensible way at any moment of time and, as mentioned, in an unpredictable environment. Note that in many similar robots the number of free DoFs is artificially reduced by a central controller (e.g., an explicit tripod controller), which simplifies the control but, on the other hand, restricts the flexibility of the system.

As a first step—and to make it simple for our understanding, but not necessarily for the system itself—, the walker is not equipped with distance sensors like vision or acoustic sensors, but only with tactile sensors situated in the legs (and possibly the antennae) measuring contact with external objects, and with proprioceptors measuring position and velocities of joints.

The walking system to be described in the following and that has been tested to be able to control a six-legged robot (Dürr et al., 2004; Schmitz et al., 2008), is based on behavioral (and to some extent neurophysiological) studies on insects, in particular stick insects. For the purpose required here the reactive controller will also be equipped with elements forming plausible expansions. At first, we briefly describe the essentials of the earlier version, Walknet, and will then introduce the expansions in Sections Further Procedural Elements and Body Model.

**Figure 1:**
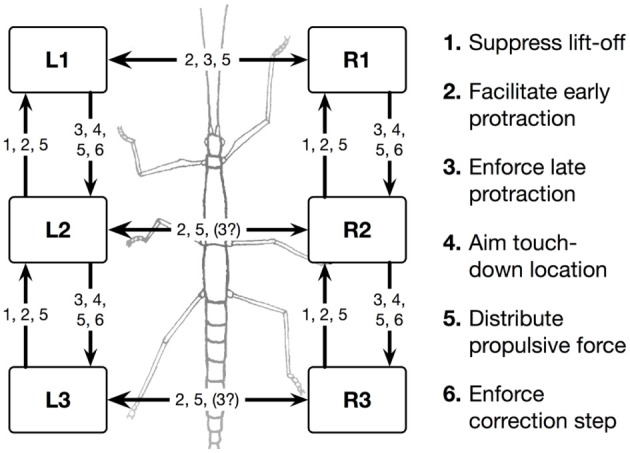
**Schema of the morphological arrangement of the leg controllers and the coordination influences (1–6) between legs**. Legs are marked by L for left legs and R for right legs and numbered from 1 to 3 for front, middle, and hind legs, respectively. The question mark indicates that there are ambiguous data concerning this influence.

**Figure 2:**
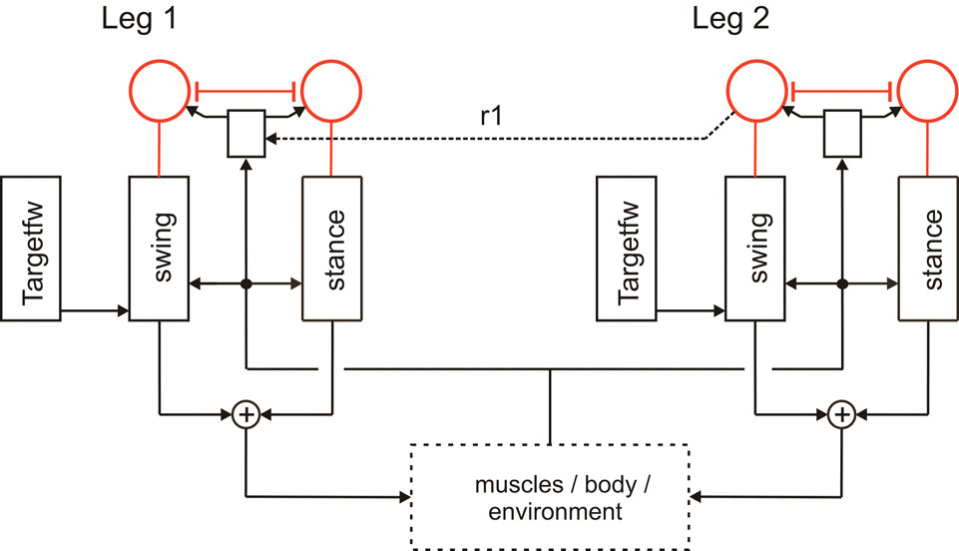
**A section of Walknet showing two leg controllers**. Each consists of a Stance-net and a Swing-net, the latter being connected with a Target-net (Targetfw). The motor output acts on the legs (box muscles/body/environment). Sensory feedback is used by the motor procedures as well as to switch between the states (red units connected by mutual inhibition). r1 represents coordination rule 1 (see Figure 1).

Forward walking in stick insects, which has already been intensively studied (Cruse et al., 1998), consists, on the leg level, of the stance movement, during which the leg maintains ground contact and is retracted to propel the body forward while supporting the weight of the body, and the swing movement where the leg is lifted off the ground and moved in the direction of walking to touch down at the location where the next stance should begin. Experiments on the walking stick insect have shown that the neuronal system is organized in a decentralized way (Wendler, 1964; Bässler, 1983; Cruse, 1990). Derived from these results, a model has been proposed in which each leg is attributed to a separate controller (Dürr et al., 2004). These single leg controllers are assumed to be situated in the thoracic ganglia [for a review see (Bässler and Büschges, 1998)]. Figure 1 sketches the approximate anatomical arrangement of the controllers and the numbering of the legs. Each controller is in charge of the behavior of the connected leg, as the controller decides which behavior is executed by this leg and in which way the joints are moved. Figure 2 shows the details of the controllers as used in Walknet for two legs (leg1, leg2, e.g., the right front leg and the right middle leg). A single leg controller mainly consists of several procedures that are realized by artificial neurons forming a local, in general, recurrent neural network (RNN). In most cases these networks consist of perceptron-like feedforward networks. These modules might receive direct sensory input and provide output signals that can be used for driving motor elements. But other modules may also provide input to a module. All these networks may be considered to form elements of the procedural memory. The two most important procedural elements in our example are the Swing-net, responsible for controlling a swing movement, and the Stance-net controlling a stance movement [Figure 2, see (Dürr et al., 2004; Schumm and Cruse, 2006) for details concerning the Swing-net, and (Schmitz et al., 2008) for Stance-net]. In addition, each leg possesses a so-called Target_fw-net (Figure 2). This net influences the Swing-net to determine the endpoint of the swing movement during forward walking. During normal forward walking the swing end-position is situated in the anterior section of the leg's range of movement (anterior extreme position, AEP). During stance the leg is moved backward until it reaches the posterior extreme position (PEP), the latter being represented by another memory element (not depicted in Figure 2).

Furthermore, a leg controller must also take into account the interaction with the other legs. Part of these interactions are mediated directly by the body and through the environment, making explicit computations superfluous [see, e.g., the local positive velocity feedback approach (Schmitz et al., 2008)]. While the physical coupling through the environment is important, it is not sufficient. In addition, the controllers of neighboring legs are coupled via a small number of channels transmitting information concerning the actual state of that leg (e.g., swing, stance) or its position (i.e., values of joint angles). These coordination rules were derived from behavioral experiments on walking sticks (Cruse, 1990; Dean, 1991a,b, 1992a,b). In Figure 1 the channels are numbered 1–6. Coordination rules 1–3 influence the length of the stance movement by influencing the transition from stance to swing movement, i.e., they change the PEP value. The Target-nets (rule 4) influence the AEP. As an example, in Figure 2 (dashed line) only one connection is shown, rule 1 (r1), which suppresses the start of a swing movement of the anterior leg (in this case the front leg, leg1) during the swing movement of the posterior leg (here the middle leg, leg2).

The local sensory influences and the coordination influences are integrated in the so-called analog selector net (Schilling et al., 2007) that decides whether a swing movement or a stance movement is performed. Activation of the Stance-net is triggered by ground contact, activation of Swing-net is triggered when the current PEP value is reached. In Figure 2 the selector net is represented by two units (marked red) which are connected by mutual inhibition, thus forming a winner-take-all network (WTA-net, for details see Section Motivation Unit Network). Activation of such a unit (between 0 and 1) controls the output of the corresponding procedure in a multiplicative way. The representation of the value for the default PEP is not depicted in Figure 2 as well as the detailed influence of the coordination rules influencing the actual PEP value (for reviews see Dürr et al., 2004; Schilling et al., 2013).

Kinematic and dynamic simulations as well as tests on robots have shown that this network can control walking in different velocities, producing different insect gaits including the continuous transition between the so called tetrapod gait and the tripod gait, negotiate curves (Kindermann, 2002), climbing over obstacles (Dürr et al., 2004) and over very large gaps (Bläsing, 2006), and coping with leg loss (Schilling et al., 2007). Thus, Walknet exhibits a free gait controller where the gaits are not explicitly implemented but emerge from a strictly decentralized architecture. Including some more recent extensions, Walknet can describe further behaviors observed in stick insects walking on variously shaped substrates (e.g., Diederich et al., 2002; Schumm and Cruse, 2006).

**Figure 3:**
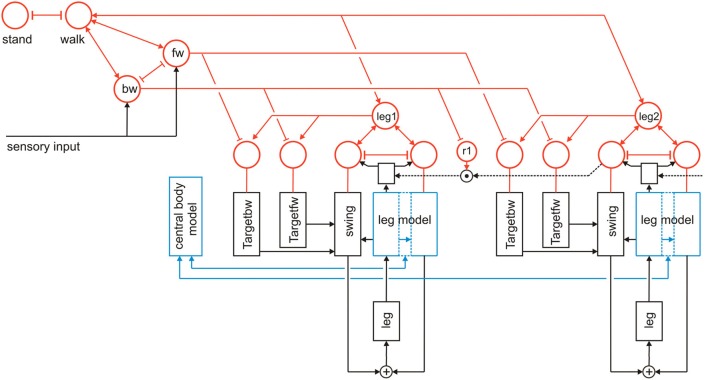
**Walknet as depicted in Figure 2, but now expanded by further procedures (Target_bw-net), a body model (blue) and a motivation unit network (red)**. The body is represented by the boxes “leg.” Note that for clarity only the target nets are depicted, but not the PEP-nets.

In the following, we will expand Walknet as illustrated in Figure 3. These expansions concern (i) the introduction of further procedural elements (Section Further Procedural Elements), (ii) a body model (Section Body Model), and (iii) a motivation unit network (Section Motivation Unit Network). The properties of the robot simulator will briefly sketched in the Appendix.

### Further procedural elements

Further procedures are required when the controller should not only be able to perform one type of behavior, for example forward walking, but also others like backward walking. Specifically, further Target-nets are introduced which can influence the corresponding Swing-net to move the leg in posterior direction as during backward walking (bw) the swing end position is in the posterior (rear) section (Figure 3, Target_bw-net). Correspondingly, the default end position of the stance movement is represented by a “PEP-net,” one for forward walking and another one for backward walking. For simplicity the latter are not depicted in Figure 3. The Target_fw-net mentioned above can be realized by a two-layer perceptron (Dean, 1990). Its function is to compute the anterior target position, the leg aims at during the swing movement. The anterior target is usually situated directly behind the current position of its anterior neighboring leg. The computation therefore represents the inverse kinematics. Alternatively, and this version is used in the simulation shown here, the Target-nets and the PEP memories are realized as a three-unit RNN with bias, representing a static memory element storing the corresponding target position. For backward walking in addition the corresponding coordination rules are required but not depicted in Figure 3.

### Body model

**Figure 4:**
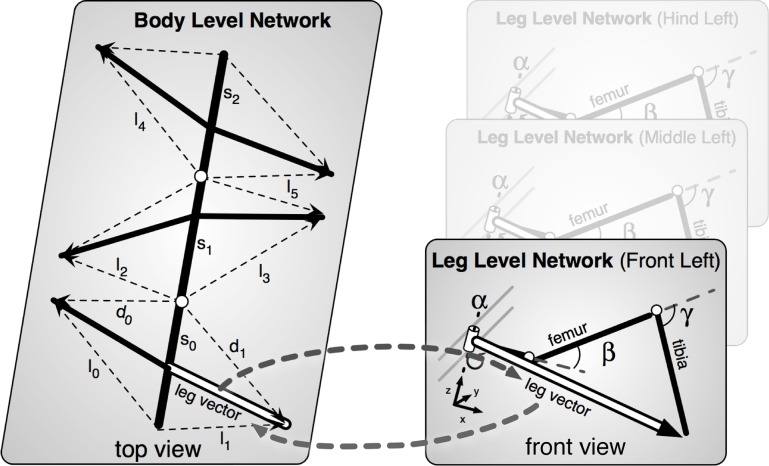
**The body model. Left**, the upper, abstract level representing the body; **Right**, three of the six networks representing the legs.

A second important expansion concerns the construction and implementation of a body model. This body model is represented by a specific RNN (Schilling, 2011) and has a modular structure (Schilling et al., 2012). It consists of six networks each representing one leg. These modules are connected on a higher level forming a seventh network representing the whole body. This network represents the central body and the legs in an only abstracted form. In Figure 3 the elements of the body model are marked in blue. Thus, the body model is represented by a modular structure which, as it is constructed as a RNN, at the same time comprises a holistic system [Figure 4, for details concerning the body model see (Schilling, 2011; Schilling et al., 2012)].

In normal walking the body model is used for controlling stance movements. It is used in forward and backward walking as well as in negotiating curves and provides joint control signals to the system. Sensory data are fed into the body model. Due to its holistic structure the body model integrates redundant sensory information and is able to correct possible errors in the sensor data (Schilling and Cruse, 2012). As will be sketched in the Discussion, due to its ability of pattern completion, this model can also be used as a forward model (Schilling, 2009). Therefore, the model allows for prediction, too.

The function of the body model is to mediate the coupling between the single leg vectors (Figure 4). During a movement these vectors have to be moved in a coherent way. While the body moves to the front, the feet should stay on the same place on the ground, i.e., the relative position between the feet must not change. As the model mirrors the 22° of freedom of the insect body the task is underdetermined and therefore this is still a hard problem and a unique solution is not directly computable (Schilling and Cruse, 2012).

**Figure 5:**
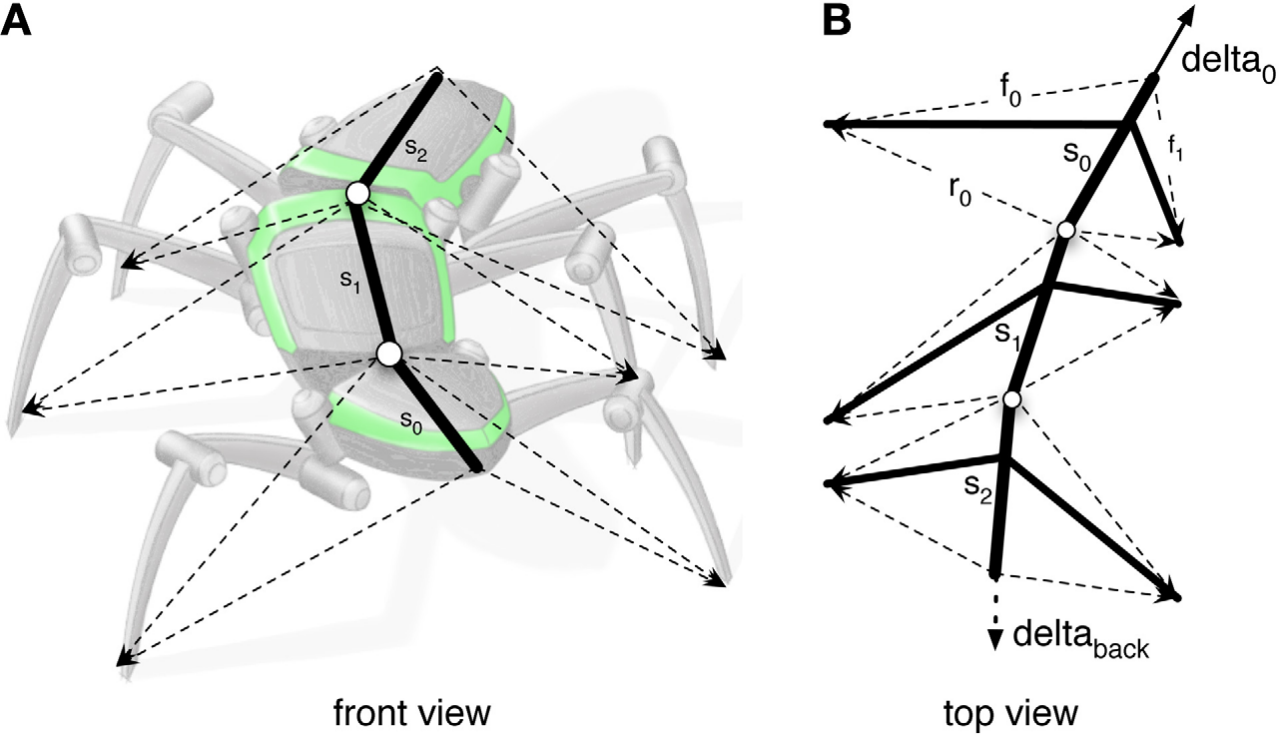
**(A)** Illustrates how the body model is attached to the body of robot Hector. **(B)** shows the vectors delta_0 and delta_back pulling the model in forward or backward direction, respectively.

As a solution, we apply the idea of the passive motion paradigm to this problem (von Kleist, 1810; Mussa Ivaldi et al., 1988; Loeb, 2001). Like a simulated marionette puppet (Figure 5B), the internally simulated body is pulled by its head in the direction of desired body movement (Figure 5B, delta_0), provided, for example, by a vector based on sensory input from the antennae (Dürr and Schütz, 2011) or, if available, by visual or acoustic input (Figure 3, sensory input, Figure 5B, delta_0, delta_back). As a consequence, the stance legs of the puppet move in an appropriate way. The changes of the simulated joint angles as well as the body joints can be used as commands to control the actual joints. If such a body model is given that represents the kinematical constraints of the real body, we obtain in this way an easy solution of the inverse kinematic problem, i.e., for the question how the joints of legs standing on the ground have to be moved in concert to propel the body (for details and application for the control of curve walking see Schilling et al., 2012). In this case, the positive feedback input given to Stance-net, as was used in the earlier version (Schmitz et al., 2008) is not anymore necessary (although application of both influences may be sensible). The Stance-net can therefore be considered to only consist of Integral Controllers, one for each leg joint to which the body model provides the reference inputs.

## Motivation unit network

To allow the system to select autonomously between different behaviors as for instance standing and walking, or forward and backward walking, Walknet is expanded by introduction of a RNN consisting of so-called motivation units (Figure 3) as has been done for Navinet (Cruse and Wehner, 2011; Hoinville et al., 2012). The units used here are artificial linear summation neurons with a piece-wise linear activation function showing lower and upper limits of 0 and 1, respectively.

The function of a motivation unit as applied here is to control to what extent the corresponding procedural element contributes to the behavior. To this end, these units influence the strength of the output of this network (in a multiplicative way). In an earlier version (Figure 2, Schilling et al., 2007) such a network containing only two motivation units has been applied to control the output of Swing-net and Stance-net. In this network each motivation unit is reinforcing itself, whilst the two motivation units are mutually inhibiting each other forming a WTA-net. Due to this competition, only one of the two behaviors is active most of the time. This decision is influenced by sensory signals acting on the motivation units. When the leg touches the ground at the front during a swing movement, the ground contact causes switching to the stance movement. When the leg reaches the PEP during stance, swing is initiated. Introduction of motivation units was inspired by Hassenstein (1983) and Maes (1991) and is based on the finding of Schumm and Cruse (2006) according to which there are indeed variable motivational states for individual procedures, in this case the swing controller.

Here we expand this network in two ways (Figure 3). First, each procedural element will be equipped with a motivation unit. This means that not only Swing-net and Stance-net, but also all Target-nets and all PEP memories as well as the leg coordination channels have an own motivation unit. Motivation units for Target-nets and PEP-nets are required to select between different target positions for forward walking and backward walking. The motivation units influencing the coordination rules are motivated by Dürr (2005), and Ebeling and Dürr (2006), who showed that coordination influences can be modulated (e.g., during curve walking).

Second, motivation units can also be used to influence other motivation units via excitatory or inhibitory connections. This is illustrated in Figure 3. Units which belong to the procedural nets controlling the six legs (only two legs are depicted in Figure 3) show mutual positive connections to a unit termed “walk” in Figure 3. This unit serves the function of arousing all units possibly required when the behavior walk is activated. Neurophysiological grounding of such an influence is given by Büschges (1998): when walking is started, the basic potential level in a number of relevant neurons is increased. In addition, following earlier authors (see Discussion) we introduce units “forward” and “backward” to activate procedures required for forward or backward walking (Figure 3, fw, bw), respectively, by selecting specific Target-nets and PEP-nets. It is only indicated in this figure that the unit “walk” may be coupled via mutual inhibition to other units that stand for different behaviors like, for example, standing still (unit “stand”). However, the corresponding procedures are not depicted (for a further expansion see Figure 10). Apart from the units fw and bw, it is also not shown that these “higher-level” motivation units, as is the case for the motivation units of Swing-net and Stance-net, may receive direct or indirect input from sensory units that influence the activation of a motivation unit.

As illustrated in Figure 3, this at first sight hierarchical structure is in general not forming a simple, tree-like arborization. As indicated by the bi-directional connections, motivation units form a RNN coupled by positive (arrowheads) and negative (T-shaped connections) influences (for details concerning the weights used see the Appendix). This structure may therefore be better described as “heterarchical.” The combination of excitatory and inhibitory connections form a network that can adopt various stable attractor states. Some of these motivation units are coupled by local winner-take-all connections. This is true for the Swing-net and Stance-net of each leg, as well as for the motivation units for forward and backward walking. Thereby, a selection of one of the available Target-nets and of the PEP-nets is possible. Excitatory connections between motivation units allow for building coalitions. As depicted in Figure 3, there are different overlapping ensembles. For example, all “leg” units and the unit “walk” are activated during backward walking and during forward walking, but only one of the two units termed “fw” (forward) and “bw” (backward) and only some of the targeting modules are active in either case. This architecture can produce various stable attractor states or “internal states” (see Appendix for details). Such a state protects the system from responding to inappropriate sensory input. For instance, as a lower-level example, depending on whether a leg is in swing state or in stance state, a given sensory input can be treated differently: stimulation of a specific sense organ leads to a Levator reflex (when hitting an obstacle the leg is briefly retracted and then lifted) when in swing, but not during stance (see Figure 5 from Dürr et al., 2004). Correspondingly, internal states can be distinguished on higher levels, as for example walking, standing still or forage (in the case of Navinet).

## Results

As this article is focused on demonstrating the structure and the functioning of the heterarchical network in cooperation with the decentralized procedural memory, we will not report on a detailed quantitative evaluation of the functional properties of the walking system as has been done by Kindermann (2002). Instead, we show six examples of walking situations for which various combinations of active motivation units are required (five cases for forward walking, one case for backward walking) as are different walking velocities, “uncomfortable” starting configurations, or curve walking. The behavior is illustrated by plotting the footfall pattern, i.e., for each leg the state swing (black) or stance (white) over time, to illustrate the temporal structure of the gaits. In addition, in the supplement we provide videos showing the behavior of the robot, the temporal development of the footfall patterns, as well as the temporal sequence of the internal states, represented by the activation of all motivation units.

During forward walking, continuously active units are walk, fw (forward), all six leg units and the units of the corresponding Target-nets and PEP-nets. More or less regularly alternating are the Swing and Stance units of the six legs.

### Straight walking

**Figure 6:**
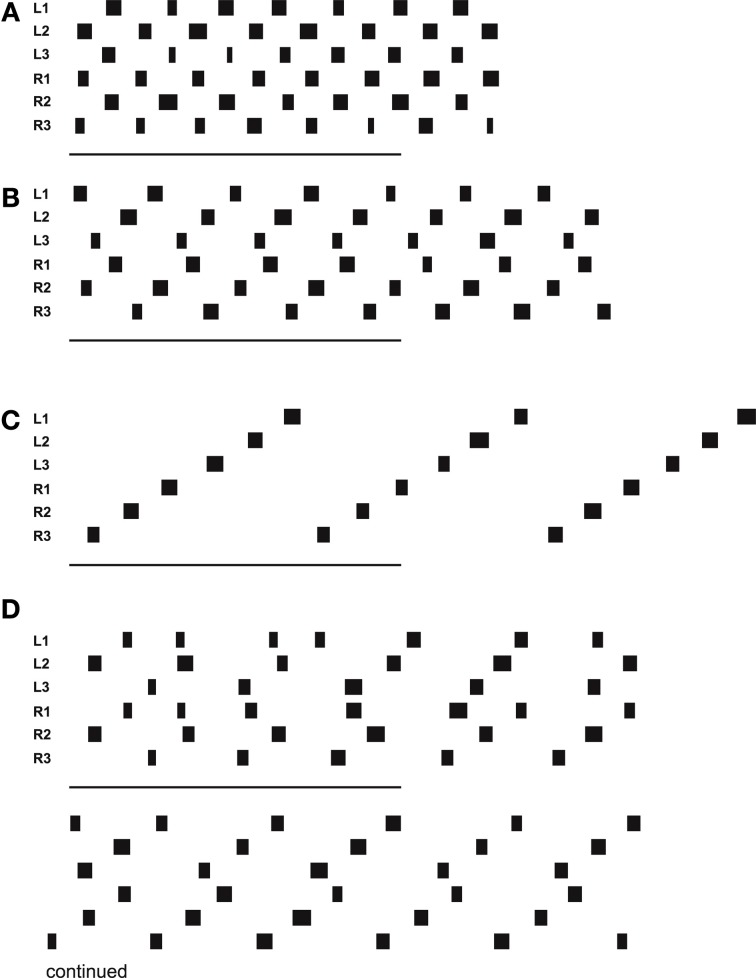
**Simulated Hector walking straight with high (A), middle (B), and low (C) speed, leading to tripod gait, tetrapod gait and wave gate, respectively. (D)** shows a walk starting from uncomfortable starting configuration. Starting position (in m, origin is position of coxa) for **(D)**: L1: 0.15, R1: 0.15, L2: -0.1, R2: -0.1, L3: -0.02, R3: -0.02. Black bars indicate swing movement of the respective leg: left front, middle and hind leg, right front, middle and hind leg, from top to bottom. Abscissa is simulation time. The lower bars indicate 500 iterations corresponding to 5 s real time.

In forward walking a vector (Figure 5B, delta_0), which might be provided by tactile input from the antennae or by visual input, is pulling the body model straight forward. Velocities are given as a dimensionless number (relative velocity v_rel = swing duration/stance duration). Figure 6A (Movie S1) shows high velocity walking (v_rel = 0.5) corresponding to what is usually called tripod gait (at least three legs are on the ground at any time). Figure 6B (Movie S2) shows a walk with lower velocity (v_rel = 0.4), usually called tetrapod (at least four legs are on the ground at any time). Figure 6C (Movie S3) shows very slow walking (v_rel = 0.15), sometimes termed wave gait. Note that, like in the insects, there is no clear separation between these “gaits.” Rather, examples shown in Figure 6 are taken from a continuum which depends on one control parameter, the velocity.

Figure 6D (Movie S4) shows a walk (v_rel = 0.3) starting from a difficult starting configuration (see legend). In this case, contralateral leg pairs started with exactly the same leg position. This leads to a gallop-kind stepping pattern (see the first three steps). A symmetry-break occurs due to minor irregularities in the ODE simulation (e.g., short slipping of a leg). After another about three to four steps, a stable wave gait pattern can be observed (Videos for all example walking cases are provided as supplementary material.).

### Negotiating curves

**Figure 7:**
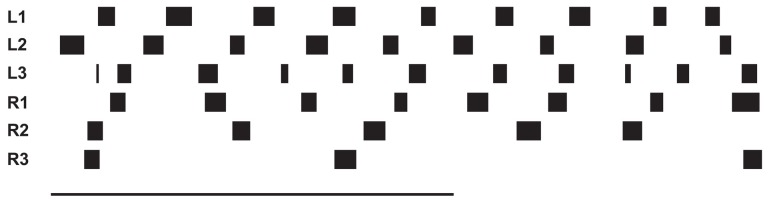
**Simulated Hector walking a turn to the right using the internal body model**. The internal body model is constantly pulled to the front and the right. The complete run shown corresponds to a turn of about 180°. Starting positions (in m, origin is position of coxa): L1: 0.20, R1: 0.05, L2: -0.04, R2: -0.14, L3: -0.02, R3: -0.22; for further explanations see Figure 6.

**Figure 8:**
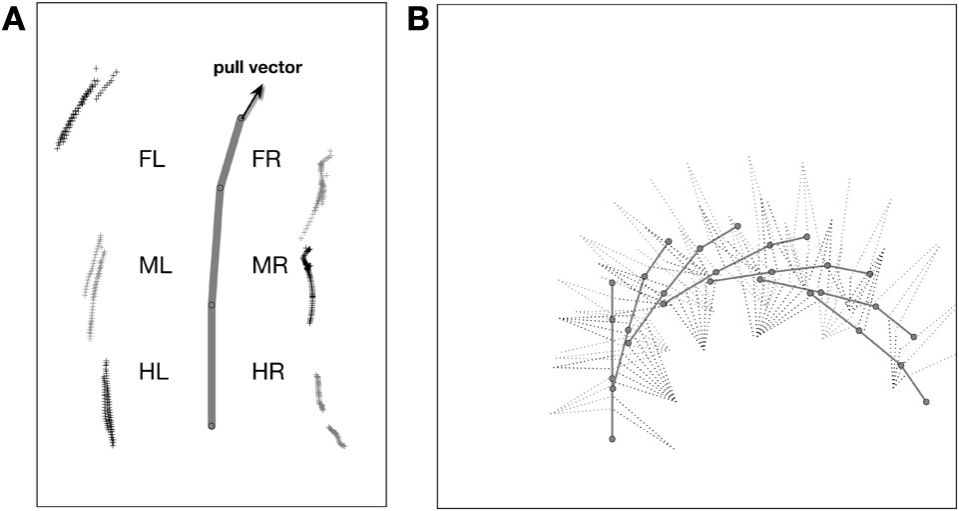
**Curve walking of the model**. In **(A)** the movement of the leg tips during the stance movement is shown with respect to the last body segment. **(B)** shows snapshots of the movement over time. Every second of simulation time the body posture is shown, the postures of the legs in stance are shown four times each second.

Still in forward mode, the body model can also be used for walking in curves which leads to another kind of leg coordination. Only the pull vector acting on the body model has to be adjusted and has to point in the direction the agent should walk to. As mentioned, the pull vector may be provided by signals from the antennae or via visual input. The body model is pulled (at the front of the first segment, Figure 5B, delta_0) into this direction and all standing legs as well as the body segments are following (Schilling et al., 2012). Figure 7 (Movie S5) shows a simulation run, where the body model is pulled to the front and the right by an angle of 12°. Velocity is set to v_rel = 0.4, i.e., no different velocities are required for right and left legs in contrast to the simulation of Kindermann (2002). We also did not change the nominal AEP and PEP values in contrast to the behavior observed in the insects (Jander, 1985; Dürr, 2005; Dürr and Ebeling, 2005; Rosano and Webb, 2007; Gruhn et al., 2009). As has been shown by these authors, during swing, the legs, in particular the front legs, target sideways, which may even allow these animals to turn on the spot (Cruse et al., 2009b). Nonetheless, the simulated agent can negotiate curves as tight as a radius of about one body length (distance between front leg coxa and hind leg coxa, 578 mm, see Figure 8B) compared to the approach of Kindermann (2002) whose tightest curves had a diameter of about three body lengths. During the sequence depicted in Figure 7, a curve of about 180° has been negotiated. In the simulation, inner legs show much smaller stance velocities and smaller step amplitude compared to the outer legs. As can be observed in Figure 7, the inner hind leg shows much smaller step frequencies and, depending on the radius of the curve, may even keep staying on the ground during the complete turn (not shown). Both results compare to those observed in insects (Dürr, 2005; Rosano and Webb, 2007; Gruhn et al., 2009).

Figure 8A Illustrates the movement of the leg tips during stance in a top–down view using as coordinate system that of the last body segment. Shown is at least one stance phase of each leg over the time of 2 s simulation time after the curve walking has been initiated for 6 s This means, that initialization of the curve has been completed and the behavior is now in a stable execution state. This is also shown by the stable orientation of the body segment angles [first segment joint has a mean value of 15.0° (std. ± 1.27), second segment joint has a mean value of 4.8° (std. ± 0.35)]. The outer legs perform faster movements during stance than do inner legs (see different distances of symbols). The outer front leg is showing movements far away from the body, mostly because the body is pulled away from its footpoint. In Figure 8B the movement is shown as a sequence, emphasizing the tightness of the curve. Here, every second of simulation time the body posture is shown and the leg postures are given for standing legs in addition four times each second. Figure 8B includes the initiation of curve walking (see also the video provided as supplementary material).

### Backward walking

To trigger backward walking, the motivation unit for backward walking (Figure 3, bw) has to be activated. In animals, this behavior can be elicited by tactile stimulation of both antennae (Graham and Epstein, 1985). Motivation unit bw activates the corresponding Target-nets and PEP-nets. Activation of this motivation unit additionally sets the forward pull vector to zero, and activates a displacement vector being attached to the back of the last segment, thereby pulling the model backwards (Figure 5B, delta_back). Furthermore, the leg coordination rules required for backward walking are switched on (here we used a mirror image version of the rules used for forward walking). As in straight forward walking or curve walking, this vector is assumed to be provided by appropriate sensory input.

**Figure 9:**
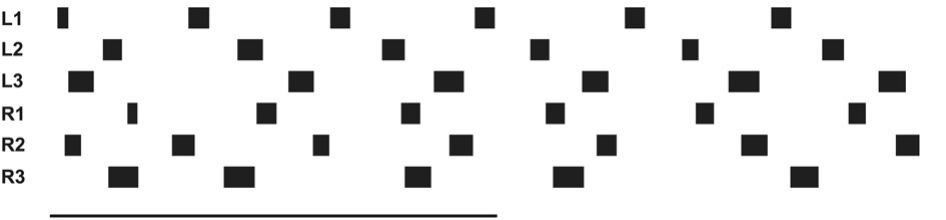
**Simulated Hector walking backwards**. Starting positions (in m, origin is position of coxa): L1: 0.20, R1: 0.05, L2: -0.04, R2: -0.14, L3: -0.02, R3: -0.22; for further explanations see Figure 6.

In the case of backward walking (Figure 9, v_rel = −0.4, Movie S6) the attractor states of the motivation unit network are characterized by the continuously active units walk, bw (backward), all six leg units as well as the units of the corresponding Target-nets and PEP-nets required for backward walking. As in forward walking, Swing-nets and Stance-nets of the six legs show variable activation patterns, which, in backward walking, also result from the different coordination rules applied. The corresponding video further outlines the details of the behavior.

## Discussion

We describe a novel architecture that can be used to control an autonomous robot and is based on earlier approaches, Walknet and Navinet. The neural controller Walknet, as described earlier (e.g., Dürr et al., 2004; Bläsing, 2006; Schmitz et al., 2008; Schilling et al., 2013), represents a typical case of an embodied controller: the network is able to control the movement of a hexapod walker in unpredictably varying environments without relying on other information than available using the given mechanosensors. This is possible because the body and properties of the environment are crucial elements of the computational system—the system is embodied not only in the sense that there is a body (i.e., that there are internal states being physically represented), but in the sense that the properties of the body (e.g., its geometry) are required for computational purposes. Exploiting the loop through the world (including the own body) allows for a dramatic simplification of the computation. In the version being expanded by an internal body model, too, control of DoFs does not result from explicit specification by the neuronal controller, but results from a combination/cooperation of the neuronal controller, the internal body model and the coupling via the environment.

The procedures forming the decentralized controller are basically arranged in parallel, i.e., obtain sensory input and provide motor output, but there are also procedures that receive input from other procedures and, as a consequence, procedures that provide output to other procedures. Application of such a decentralized architecture helps to solve the flexibility—automaticity dilemma. Modules have a fixed function, but can work together in a flexible way to solve difficult tasks. In the case described here this is realized by a system that, based on studies of insect behavior, has been designed to control hexapod walking but is also able to climb over very large gaps (Bläsing, 2006).

Flexibility of the system is improved by introduction of the motivation unit network (Section Motivation Unit Network), which allows to integrate additional behaviors in the process of behavioral selection. The organization of this network is especially designed to allow for competitions on different levels, in this way forming different clusters of units. For example, the competition on a leg level selects swing and stance movements, while, on a more global level, the walking direction or other behaviors different from walking can be selected. Activities of these motivation units not only allow for selection of behavioral elements, but also provide a context according to which specific sensory inputs are attended or not. In this sense, the motivation unit network can be considered to be a system allowing for controlling attention. This property is more obvious in another case, where the architecture proposed here has successfully been applied for controlling insect navigation (“Navinet,” Cruse and Wehner, 2011; Hoinville et al., 2012). In this task the animals are able to select visiting one of a number of food sources learned, and to decide between traveling to the food source or back home. Of particular interest is here that a desert ant and also Navinet attend known visual landmarks only in the appropriate context, i.e., depending on the food source it is actually traveling to. In this way, the system controlled by the motivation network allows for selective attention. As Navinet provides walking direction as output, both Navinet and Walknet can directly be combined, whereby the output of Navinet controls the pull vector of the body model.

Introduction of motivation units does not impair the behavior of the basic Walknet structure, because during walking in one direction (forward or backward) all motivation units maintain their activation values except for the Swing and Stance motivation units. Only the latter change their activation dynamically and do this in the same way as is the case in the earlier Walknet versions. Therefore, all properties of Walknet concerning forward walking are inherited in the version expanded by motivation units.

**Figure 10:**
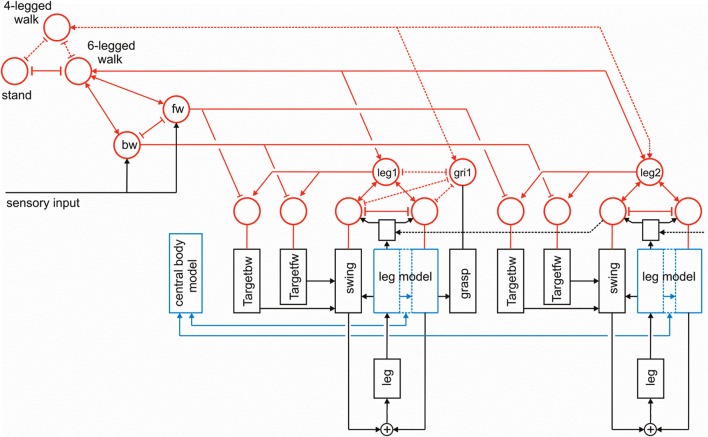
**The same network as shown in Figure 3, but now expanded by a procedure that allows for switching between 6-legged walk and 4-legged walk, where front legs (here leg 1) can be controlled by another procedure (“grasp,” output not shown) via a motivation unit “gri1.”** The additional connections required are marked by dashed lines.

Although the procedures as such are essentially arranged in parallel, the motivation network provides connections between the modules that form a dynamical heterarchy. In contrast to Jenkins and Mataric (2003), for example, who discuss a three level structure (motor level, skill level, task level), our architecture does not imply such a strict separation in levels. Rather, any combination of modules might, in principle, be possible in this architecture. Furthermore, this architecture is very flexible as it easily allows for later expansions to represent not only six-legged walking, but, for example, four-legged walking where both front legs could be used as grippers. How this could be done is illustrated in Figure 10. As depicted in this figure, to this end the front leg controller, in the figure leg 1, is equipped with a parallel procedure termed “grasp,” that is controlled by an own motivation unit (Figure 10, gri1). This motivation unit is activated if the unit “4-legged walk” is active, which in turn inhibits the unit “6-legged walk.”

**Figure 11:**
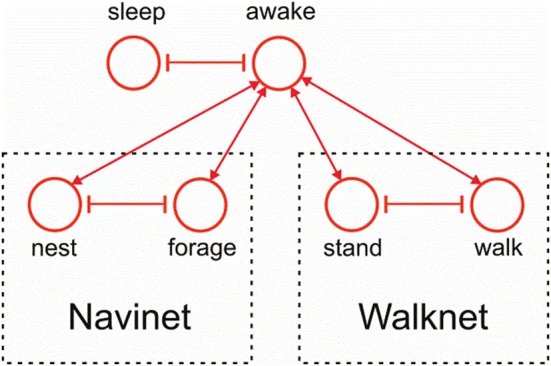
**Combination of the motivation unit network of Walknet as depicted in Figure 3 and of Navinet (Cruse and Wehner, 2011, Figure 1)**.

Figure 11 indicates how the motivation unit network of Navinet (Cruse and Wehner, 2011; Figure 1) and that of the Walknet version depicted in Figure 3 can be combined. To this end, we introduce a higher layer consisting of two motivation units “sleep” and “awake,” which are then connected to the uppermost layer of Walknet (Figure 3), units “stand” and “walk,” and to the uppermost layer of Navinet, units “nest” and “forage.”

Our network provides an example showing that concatenation of modules required for control of complex behavior does not necessarily require explicit coding, but may emerge from local rules and the coupling through the environment. The heterarchical structure used in the expanded version of Walknet and in Navinet comprises a simple realization of “neural reuse” as proposed in Anderson's massive redeployment hypothesis 2010.

The network, as described, consists of a “hard-wired” structure, i.e., the weights connecting the artificial neurons are fixed. Nevertheless, the system is able to flexibly adapt to properties of the environment. However, there may also situations occur in which the controller runs into a deadlock. Think for example of the situation in which, during forward walking, by chance all legs but the right hind leg are positioned in the frontal part of their corresponding range of movement, whilst the right hind leg is positioned very far to the rear. When this leg starts a swing movement, the body may fall backward as the center of gravity is not anymore supported by the legs on the ground. Such a “problem” might be signaled by specific sensory input, for example a specific load distribution of the legs. To find a way out of this deadlock, a random selection of a behavioral module not belonging to the actual context, in our case forward walking, may help. A possible example might be a backward step of the right middle leg. Such a backward step of the middle leg would make it possible to support the body, then allowing the hind leg to start a swing. However, in our controller, backward steps are only permitted in the context of backward walking. How might it be possible for the system to find such a solution nonetheless?

**Figure 12:**
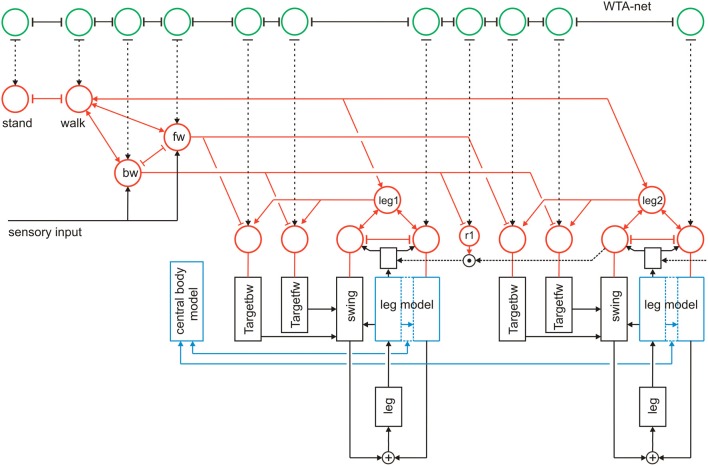
**Walknet as depicted in Figure 3, with a body model (blue) and a motivation unit network (red), but now expanded by a further layer (WTA, marked green, not all connections are depicted)**. The body is represented by the boxes “leg.”

In Figure 12 we briefly illustrate a simple expansion allowing the system to search for such a solution (Schilling and Cruse, 2008). A third layer, essentially consisting of a recurrent WTA-net, is arranged in such a way that each motivation unit has a partner unit in the WTA-net (Figure 12, green units). The WTA units might be activated by various “problem detectors” not depicted in Figure 12. Motivation units activated in the actual context inhibit their WTA partner unit (T-shaped connections in Figure 12). Thus, a random activation of the WTA-net will, after relaxation, find one of the currently not activated modules. The WTA unit winning the competition can then be used to activate its partner motivation unit and thereby trigger a new behavior that can be tested for being able to solve the problem. In this way, realizing a special type of top down attention, the network has the capability of following a trial-and-error strategy.

As has been proposed (Schilling and Cruse, 2008) a further expansion of the system may permit to use the body model instead of the real body to test the new behavior via “internal trial-and-error” whilst the motor output to the real body is switched off. To this end, switches have to be introduced allowing the motor output signals to circumvent the real body and being passed directly to the body model (not depicted in Figure 12). Only if the internal simulation has shown that the new trial provides a solution to the problem, the behavior will actually be executed. McFarland and Bösser (1993) define a cognitive system in the strict sense as a system that is able to plan ahead, i.e., to perform internal simulations to predict the possible outcome of a behavior. Therefore, the latter expansion would, according to McFarland and Bösser, make the system a cognitive one (for details see Schilling et al., 2013).

Furthermore, inspired by Steels and Belpaeme (2005); Steels (2007), the possibility to expand the network by a forth layer, that contains specific procedures, namely networks that represent verbal expressions has been discussed by Cruse (2010). These “word-nets” may likewise be used to utter or to comprehend the word stored. The underlying idea is to connect each word-net with a unit of the motivation network of which it carries the meaning (e.g., the word-net “walk” should be connected with the motivation unit walk), thereby grounding the symbolic expression (Cruse, 2010). Interestingly, Jenkins and Mataric (2003) draw an analogy between the structure for what they call a “motor vocabulary” to linguistic grammar or “verb graphs,” a property that is reflected in our network.

**Figure 13:**
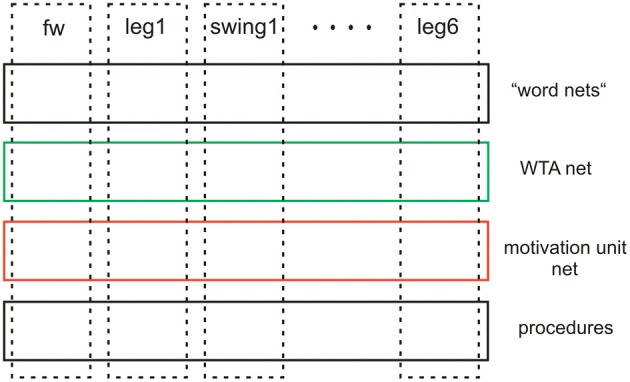
**Schematic showing the horizontal and columnar arrangement of the modules used by the architecture proposed**.

Although the latter two levels (WTA-net and word-nets) are still quite speculative as they have not yet been tested, together with the two lower layers they illustrate the principal idea of this architecture (Figure 13). Horizontally arranged modules (procedures, motivation units, WTA neurons and procedures for words), are ordered in the horizontal layers in such a way that the corresponding elements in the different layers appear in a vertical order, leading to modules arranged in a columnar fashion (Figure 13, dashed rectangles). Addressing this columnar structure does not mean that each lower level procedure or each motivation unit has to have a partner in the upper layers, but only means that such connections are in principle possible. Similarly, not every unit or procedure in the upper layers necessarily has a partner procedure in the lowest layer. As a column in this architecture is characterized by a motivation unit, we name our architecture MUBCA.

Interestingly, Schack and Ritter (2009) and Schack (2010) have, too, proposed a four layer model to describe an architecture for the control of complex movements, which makes a comparison tempting. The four layers of this model are characterized as (i) sensorimotor control, (ii) sensorimotor representation, (iii) mental representation, and (iv) mental control. Although this architecture is settled at a more abstract level of description, our procedural layer might well be compared with Schack's sensorimotor control layer. Further, our motivation network has relations to Schack's sensorimotor representation layer (ii). Schack's layers (iii) and (iv) are not directly comparable, as “mental” is defined in his approach by consciously accessible, an aspect not considered here. Nonetheless, our WTA layer may be related with Schack's uppermost layer (iv), the mental control layer. Our uppermost layer might better be related with the mental representation layer of Schack's model, i.e., his layer (iii). This layer is characterized by containing the so called basic action concepts, behavioral elements to which verbal expressions can be assigned. So, there appear to exist some interesting relations between Schack's model and ours, but obvious differences can be observed in detail.

Another proposal, the DAC architecture developed by Verschure and cooperators (review Verschure, 2012), is at first glance formally very similar to our approach, as it consists of four layers and (three) columns. The four layers show some relation to our layers. DAC differentiates between soma (body including sensors and actuators) and three layers characterizing the brain, the reactive layer, the adaptive layer and the contextual layer. The reactive layer roughly corresponds to our procedural layer, the adaptive layer controls (classical and operant) learning, not addressed in our approach. The contextual layer has some relation to our motivation unit network. The contextual layer of the DAC architecture also contains memory elements (STM, LTM) in contrast to our system where these memories are only stored in the lower, procedural layer. The motivation units only contribute to selecting the different procedures containing the memory content. The three columns, however, are quite different from the columns used in our architecture. They concern (i) the state of the world, (ii) the state of the self, and (iii) control of action. In our network, these functions are implicitly embedded in the different layers making our architecture much simpler. Learning is, in our model, foreseen to be implemented as explorative learning (Schilling et al., 2013) and will be realized by a cooperation of our third layer (WTA net) and the second layer (motivation unit network).

Our architecture follows the concept of D'Avella et al. (2003) assuming that natural motor patterns are constructed by combination of discrete elements (“modules,” “motor programs”). To simplify the simulation, we assume that different modules are constructed of separate, non-overlapping neuronal elements. The situation might, however, be more complicated. As reviewed by Briggman and Kristan (2008), “morphologically defined circuits could be reconfigured into many distinct functional circuits […] generating recognizable discrete behaviors.” To cope with this case, for example Tani et al. (2004) studied how a number of different behaviors can be represented by different states of one RNN. A similar question can be asked with respect to the motivation units. Are they better described by individual units or do they form a distributed structure? Tani (2007) showed interesting studies of models, where not individual motivation units are responsible to modulate a given behavior. In contrast, a higher level RNN adopts different attractor states which as such influence the properties of lower level RNN. As, however, distributed systems containing recurrent connections appear to be more difficult to be stabilized, we have chosen to deal with distinct modules on the lower level and single units at the higher level. This is in particular helpful when dealing with a large number of modules and aiming to control relatively complex behaviors as is the goal of Walknet and Navinet.

Two further approaches should be mentioned that address the problem of how various sensory input can be used to select between different behaviors. The architecture of Arena et al. (2009) essentially consists of a number of “basic behaviors” (e.g., phonotaxis, phototaxis, obstacle avoidance) and a “representation layer.” The former compare with our procedures, the latter is related to our motivation unit network layer. Whereas the motivation unit layer comprises a simple, sparsely coded Hopfield-like network forming a decentralized structure with local, dedicated sensory inputs for some of the units, the Representation Layer of Arena et al. (2009) is quite different as it receives all sensory inputs to form a “central representation of the actual environmental situation” being represented by a Turing pattern. The Turing pattern emerges from a structure consisting of sensory neurons and two layers of Reaction-Diffusion Cellular Non-linear Networks (CNN) which, through temporal dynamics leads to static Turing patterns. The attractors represented by these patterns depend on the actual activation of the sensory cells. An additional selector network computes, after learning is finished, the relative contribution of the different basic behaviors to the overall behavioral output. A comparable selection process is, in our system, performed directly by the motivation unit network, that allows for parallel activation of different procedures except for those that are directly connected via inhibitory weights. As an important functional difference to our approach, learning plays a crucial role for the system developed by Arena et al. (2009), requiring a more complex architecture. As in our system no (online) learning is taking place, the network can be dramatically simplified and allows a simple way of finding sensible combinations of procedures. A central representation of the actual situation given by the current sensory input is not required.

An, on a general level, similar architecture, that transforms sensory input data into a vector that then is used to drive the single behaviors is given by Steingrube et al. (2010). Not counting on details, the representation layer of Arena et al. (2009) is here replaced by a network showing chaotic properties. The neuronal chaos controller requires a preprocessor and a postprocessor for sensorimotor mapping able to select between 11 different basic behaviors, whereby at least six of them are considered as “typical walking patterns emerging in insects.” Although this solution is from a mathematical point of view quite interesting, the biological grounding is not well justified. The different gaits used should not be interpreted as discrete “basic behavioral patterns.” Rather, as discussed earlier, these “gaits” are arbitrarily selected patterns out of a continuum and should therefore be considered as one basic behavior. Furthermore, although being claimed as allowing for fast switching between behaviors, the chaos controller is slower than our very simple motivation unit network which requires one or at the most two iteration for finding another attractor.

Both approaches are characterized by application of centralized structures to solve the problem of combining a large amount of sensor data and use this information to control various different behaviors. As an alternative, here we propose a decentralized solution. Our approach of using individual motivation units is supported by evidence for the existence of discrete neurons on the higher level, at least for invertebrates. For example, Briggman et al. (2005) show that in the leech a specific neuron drives crawling, while experimental inhibition of this neuron supports swimming. In Aplysia feeding behavior, a “command-like neuron” influenced by motivational states normally elicits ingestion behavior. After experimental application of a specific neuropeptid, this neuron elicits egestive behavior, thus deciding between two mutually excluding behaviors (Jing et al., 2007). Briggman and Kristan (2008) reviewed further examples including units releasing neuromodular substances.

Application of motivation units for controlling the selection of different procedures arranged in parallel have been introduced by Maes (1991, see also Hassenstein (1983), both inspired by K. Lorenz). Maes also included connections between motivation units that allow to control temporal relationships between the procedures, a property not applied here. Instead, we introduced a heterarchical structure to allow for the selection of various combinations of modules. Simple, non-heterarchical structures have already been applied in Walknet to select between procedures (Swing—Stance, Schilling et al., 2007) and between higher-level states (forward—backward, (Schilling and Cruse, 2008), then called “distributor net”). In the form of so-called command neurons the selection between forward walking and backward walking has already been applied by Ayers and Davis (1977). Similarly, mutually inhibitory units used to decide between forward and backward walking have also been used in a model of Tóth et al. (2012). In both cases, the units controlling forward or backward walking are directly connected with the motor units on the muscle level, in contrast to our approach. More generally, these models are based on strictly hierarchical structures what makes it difficult to use individual modules in other ways than allowed in the actual context. Therefore, these hard-wired hierarchical structures prohibit trial-and-error variations in cases of emergency. Our heterarchical system with selective access to individual modules makes this possible (Figure 12 and related text).

Although our approach has originally been derived from studying insect behavior, the neuronal architecture is purposefully abstracted from any neuroanatomical constraints given by the insect brain. Therefore, concentrating on the functional aspect and searching for some kind of minimal model, this architecture may be applied to different types of brains. Nonetheless, in the following we will briefly discuss to what extent this model may be mapped to the neuronal system of insects. Some motivation units clearly should be part of local, thoracic ganglia. These are the motivation units for swing and stance, target_fw, target_bw, PEP_fw, PEP_bw of the different legs, as well as most probably the six “leg” motivation units. Less clear is the localization of the motivation units controlling the different coordination influences acting between the legs. All other motivation units, like stand, walk, forage, (stay in) nest, forward, backward, are to be attributed to the brain of the insects.

Finally, a nomenclatural question that might be briefly discussed is whether it is sensible to attribute a higher level term as “motivation” to such simple units as used here to control microbehaviors like swing or stance, for example. Generally accepted examples for motivational states are, for instance, aggression controlling fight, or fear controlling flight. However, in our network, there is functionally no principal difference between motivation units controlling behaviors at any level of our network. Therefore, we believe it is justified to apply this term also to such lower level elements, or “microbehaviors,” like swing or stance of a leg, for example. Of course, this usage of the term motivation often applied in robotics and animal behavior, is still quite different from that used in psychology, focusing on systems with cognitive abilities. Motivation in psychology is generally considered as a (multiplicative) combination of desire and expectancy. In our case, only the aspect of “desire” is addressed.

## Conclusion

Our architecture, MUBCA, integrates often discussed properties postulated to exist in neuronal systems, as are modularity, hierarchy, cross modal influences (e.g., path integration and landmark navigation in Navinet), bottom–up and top–down attention control, i.e., selection of relevant input data establishing priorities, application of internal models for prediction, and redundant structures. Due to the fact that some central structures as the motivation unit network and the body model are realized as RNN, the complete network forms a holistic system. Therefore, this architecture can be considered as modular and holistic at the same time. As this architecture does not follow strict rules for construction, it is very flexible and can be expanded in various ways. To verify these capabilities further, our next step to do is to implement the cognitive layer as sketched in Figure 12. In parallel, all versions of the model will be tested on the physical robot Hector. The versatility of this approach is further underlined by a proposal as to how this architecture could be expanded to show properties of a mirror system and Theory of Mind (Cruse and Schilling, 2011). Recently, these authors have argued that even higher-level properties as are intention, (bottom–up and top–down) attention, volition as well as some aspects of consciousness can be attributed to a network based on this architecture. Interestingly, these properties are not explicitly implemented but appear as emergent properties of the network (Cruse and Schilling, 2013).

### Conflict of interest statement

The authors declare that the research was conducted in the absence of any commercial or financial relationships that could be construed as a potential conflict of interest.

## Supplementary material

Movie S1 **Corresponds to Figure 6A, fast walking, normal starting posture**.

Movie S2 **Figure 6B, medium walking velocity, normal starting posture**.

Movie S3 **Figure 6C, slow walking, normal starting posture**.

Movie S4 **Figure 6D, medium walking velocity, difficult starting posture**.

Movie S5 **Figure 7 curve walking**.

Movie S6 **Figure 9 backward walking**.

## Appendix

### Motivation unit network

The motivation unit network as applied here represents a recurrent neural network with piecewise linear summation units and bounded activation function (output ≥ 0 and ≤ 1). Each unit has a fixed self-activation weight [w/(w + 1)], which gives each unit the property of a first order low-pass filter with a time constant tau = w − 1 (here we used w = 3). The used weights are given in the matrix shown in Table A1. Note that all values are multiplied by (w + 1). In Table A1 several submodules can be identified. One consists of the two units Stance and Walk, the other of units Walk, Fw, Bw, leg1 … leg6. In addition, there are six leg submodules consisting of leg#, Sw# and St#, (# = 1–6). Motivation units for Target-net, PEP-net and Coordination Rules only receive top–down influence. This is done to simplify the net dynamics, but in principle recurrent connections were possible, too.

**Table A1:** **The weights required for the motivation unit network (Figure 3, marked red)**.

	**St**	**wa**	**fw**	**bw**	**leg1**	**sw1**	**st1**	**Tfw1**	**Tbw1**	**Pfw1**	**Pbw1**	**R1fw**	**R1bw**	**leg2 …**
st	W	−1												
wa	−5	W	1	1	0.2									0.2
fw		1	w	−3										
bw		1	−3	W										
leg1		1			W	1	1							
sw1					1	W	−3							
st1					1	−3	w							
Tfw1				−3	1			w						
Tbw1			−3		1				w					
Pfw1				−3	1					w				
Pbw1			−3		1						w			
R1fw				−3	1							w		
R1bw			−3		1								w	
leg2 …		1												w

Upper row and left column refer to the individual motivation units. Weights arranged in a horizontal row mark input weights of the unit shown at the left. Units marked in Figure 3 by “stand,” or “walk” are abbreviated here by “st” or “wa,” respectively. Units shown in Figure 3 as “fw,” “bw,” “leg1,” “leg2” … are shown as marked in Figure 3. Units not explicitly marked in Figure 3 are the motivation units for the swing procedure (here abbreviated by sw plus the number for the corresponding leg), the motivation unit for the stance procedure (marked by “leg model” in Figure 3) here abbreviated by st plus the number for the leg. The motivation units for the target nets are abbreviated by Tfw and Tbw for forward walking and backward walking, respectively plus the number for the leg. Correspondingly, the motivation units for the PEP nets, not shown n Figure 3, are abbreviated by Pfw and Pbw plus the number for the leg. Furthermore, motivation units for coordination rule 1 is abbreviated by R1fw and R1bw for forward walking and backward walking, respectively. All weights multiplied by (w + 1); w = 3.

Training of such a network is possible for linear and non-linear activation functions (Makarov et al., 2008; Cruse and Schilling, 2010). To obtain sparse coding, only those weights were allowed to be learned that in Figure 3 are represented by (red) connections. By this way, the different procedures controlling the six legs were kept separate which simplifies the number of attractors to be trained. For training the leg network we used four vectors characterized by activation of units (1) walk, fw, leg1, sw, (2) walk, fw, leg1, st, (3) walk, bw, leg1, sw, and (4) walk, bw, leg1, st. Application of the procedure proposed by Makarov et al. (2008) guarantees to find stable attractor solutions even for a piecewise-linear activation function showing no upper limit. However, following this procedure, stability of the network would depend on the exactness of the weight values, i.e., noise would deteriorate the stability. We therefore, made this network more stable against noisy weights by introduction of an upper limit of 1 for each activation function. In this way, the network shows a Hopfield-like structure, apart from asymmetric weights being possible. Introduction of this activation function further allows in a second step, to increase the weight values in order to decrease relaxation time. To this end, within a leg, positive weights were increased to 1, negative weights decreased to −3. The weight values of the upper motivation unit “walk” that connects all leg units, were only increased to a value of 0.2, but the inhibitory weights decreased to −5, to cope with the different number of positive (five) and negative (one, in the example of Figure 3) input channels. This two-step procedure makes the already stable trained net solution more stable with respect to noisy weight values and allows for faster relaxation. Note that the figures for the weight values given here and in Table A1 are multiplied by a factor (w + 1) with w = 3.

With the weights given in Table A1, the motivation network shows the following dynamic properties. Motivation units connected via mutual inhibition (e.g., Walk—Stand, Swing—Stance) change from zero to one (and back) within one iteration. Motivation units which receive only feedforward input (e.g., motivation units of Target-nets or PEP-nets) change their output value following a low-pass filter dynamic with a time constant of four iterations.

An important aspect is that (sensory) input driving the motivation unit has to show a transient component, i.e., has to be endowed with high-pass filter like properties. The amplitude of the first pulse is tuned by hand and depends on the value of the inhibitory weight stabilizing the WTA elements.

### Robot simulation

As mentioned in the Introduction, to test the capability of our controller, a physical robot or, as a first step, a physics engine is required that is able to simulate the robot and its interaction with the environment. The robot named Hector (Schneider et al., 2011), currently being developed, has 22 DoFs. The spatial arrangement of the joints corresponds to that found in stick insects, however, all measurements are scaled by a factor of 20. The distance between the onset of the front leg and the middle leg is 360 mm, the corresponding distance between middle leg and hind leg is 218 mm. The three leg segments have a length of 30, 260, and 280 mm (coxa, femur, tibia, respectively). For the robot, all legs are constructed identically in order to simplify the design. The nominal step length of a leg is 310 mm, i.e., about 85% of the scaled up step length as found in stick insects (Stick insects have a step length of about 18 mm which would correspond to 360 mm in the scaled robot.). The step amplitude is limited mainly because in the stick insects, different to the robot, front legs and hind legs are longer than the middle legs. Nevertheless, step amplitude is quite large compared to usual 6-legged robots.

For the simulation the Open Dynamics Engine is used as a physics engine to simulate the interaction between all the moving parts and with the environment. The simulation basically consists of a rigid body dynamics simulation engine and a collision detection engine. Within this virtual environment the robot is rebuilt and the inertias of the sub-assemblies of the robot are set according to their real complement. The geometry of the housings and the movement ranges correspond to the real robot. The joints in the real robot will consist of a joint drive coupled with elastic properties in order to model muscle-like characteristics (Annunziata and Schneider, 2012). In the computer simulation the elasticity is also incorporated into the joint actuators and corresponds to a spring-damper-system. As the nature of the friction between the legs and the environment is generally quite complex in real systems, these contacts are hard to simulate. Therefore, these interactions can only be approximated and this will lead to differences between the virtual and the real system. This makes the application of the real robotic system important. The weight of the three simulated body segments is 2000 g, each.

All simulation experiments were made on flat terrain, however, due to (in particular for fast walking) considerable up- and down movements of the body, irregular ground was mimicked. In addition, as friction is limited, eventually legs showed slipping in various directions during stance. The resulting irregularities are also visible in the footfall patterns
